# Load-distributing-band cardiopulmonary resuscitation for out-of-hospital cardiac arrest increases regional cerebral oxygenation: a single-center prospective pilot study

**DOI:** 10.1186/s13049-015-0182-3

**Published:** 2015-11-14

**Authors:** Yoshihito Ogawa, Tadahiko Shiozaki, Tomoya Hirose, Mitsuo Ohnishi, Yasushi Nakamori, Hiroshi Ogura, Takeshi Shimazu

**Affiliations:** Department of Traumatology and Acute Critical Medicine, Osaka University Graduate School of Medicine, 2-15 Yamadaoka, Suita, Osaka 565-0871 Japan; Department of Emergency and Critical Care Medicine, Kansai Medical University, 10-15 Fumizonocho, Moriguchi, Osaka 570-8507 Japan

**Keywords:** rSO_2_, Resuscitation, LDB-CPR

## Abstract

**Background:**

Despite advances in therapeutic strategies and improved guidelines, morbidity and mortality rates for out-of-hospital cardiac arrest (OHCA) remain high. Especially, neurological prognosis is one of the most important problems even though brain protection therapy for patients with OHCA has improved greatly in recent years due to the development of emergency post-cardiac arrest interventions such as mild therapeutic hypothermia, early percutaneous coronary intervention, and extracorporeal cardiopulmonary resuscitation (CPR). Recently, cerebral regional oxygen saturation (rSO_2_) has received attention as a method for evaluation of cerebral oxygenation. We have reported that conventional chest compression did not improve the rSO_2_ of cardiac arrest patients if they did not achieve return of spontaneous circulation. It is, however, unclear whether a mechanical CPR device is helpful in improving rSO_2_. The purpose of this study was to evaluate the effects of load-distributing-band CPR (LDB-CPR) on rSO_2_.

**Methods:**

In this prospective study, LDB-CPR was begun for OHCA with the AutoPulse^TM^ device on patient arrival at hospital. During mechanical CPR, rSO_2_ values were recorded continuously from the forehead of the patients. CPR for patients with OHCA was performed according to the Japan Resuscitation Council Guidelines 2010 except for using the AutoPulse^TM^ instead of manual chest compression.

**Results:**

From December 2012 to December 2013, 34 patients (mean age, 75.6 ± 12.8 years) with OHCA were included in this study. Duration of time from recognition of cardiac collapse to arrival to hospital was 31.0 ± 11.4 min. Compared with the rSO_2_ value of 38.9 ± 0.7 % prior to starting LDB-CPR, rSO_2_ values at 4, 8 and 12 minutes increased significantly after initiation of LDB-CPR (44.0 ± 0.9 %, 45.2 ± 0.8 %, and 45.5 ± 0.8 %, respectively, p < 0.05).

**Conclusion:**

LDB-CPR significantly increased the rSO_2_ of cardiac arrest patients during resuscitation.

## Background

Sudden cardiac arrest is a leading cause of death and remains a major public health problem in the industrialized world [1]. Despite improvements in resuscitation practice including the ‘chain of survival’, outcomes from out-of-hospital cardiac arrest (OHCA) remain poor [2, 3]. Especially, neurological prognosis is one of the most important problems even though brain protection therapy for patients with OHCA has greatly improved in recent years due to the development of emergency post-cardiac arrest interventions such as mild therapeutic hypothermia, early percutaneous coronary intervention, and extracorporeal cardiopulmonary resuscitation (CPR) [4]. For cerebral resuscitation, it is important to maintain cerebral oxygenation during CPR. However, only a few reports have evaluated cerebral oxygenation, and there is no effective way of maintaining cerebral oxygenation with manual CPR.

Previous reports have noted that an inadequate intraoperative value of cerebral regional oxygen saturation (rSO_2_) is a significant predictor of postoperative neurological complications after cardiovascular surgery [5, 6]. Now, rSO_2_ can be measured noninvasively in real time by near-infrared spectroscopy (NIRS). NIRS does not require vascular pulsation and can measure rSO_2_ even in patients with hypotension, hypothermia, and/or circulatory arrest. rSO_2_ on hospital arrival is a potential novel predictor of neurological outcomes at hospital discharge in patients with OHCA.

Therefore, high-quality chest compressions are emphasized by the International Liaison Committee on Resuscitation (ILCOR) [7]. Mechanical chest compression devices have been developed to assist rescuers in providing consistent high-quality compressions. A mechanical chest compression device that uses a load-distributing band (LDB) has been shown in animal and human studies to improve hemodynamic parameters over that of manual CPR [8, 9]. The results show that the ability to achieve return of spontaneous circulation (ROSC) with mechanical chest compression devices is significantly improved when compared with manual chest compressions. Therefore, the purpose of this study was to evaluate both the changes of rSO_2_ during CPR and the effects of LDB-CPR on rSO_2_.

## Materials and Methods

### Study Population

Osaka University Hospital, with one of the largest emergency departments in western Japan, services approximately 1 million people who reside in a 200-km^2^ area in the suburbs, which includes a number of high-rise condominiums and some mountainous regions. The population density is approximately 10,910 persons per km^2^, and there are 27 fire stations with a corresponding number of emergency dispatch centers. Emergency medical services (EMS) at these fire stations are provided by municipal governments. EMS personnel are trained in all aspects of advanced life support procedures for prehospital emergency care.

We performed a single-center prospective pilot study that included patients with OHCA transferred to the emergency room of Osaka University Hospital from December 2012 to December 2013. The exclusion criteria included a patient age of <15 years and OHCA caused by trauma.

The study protocol complied with the guidelines for epidemiologic studies issued by the Ministry of Health, Labour and Welfare of Japan and was approved by the Ethical Committee at Osaka University (no. 12385). Every patient received the standard care available at the hospital, and no patient received any experimental intervention. In light of these safeguards, the Ethical Committee approved this study and permitted the study to be exempt from the acquisition of oral or written consent.

### Interventions

All patients underwent conventional CPR with manual chest compressions delivered by EMS personnel with or without bystander CPR. After arriving at the hospital, the patients underwent conventional CPR with manual chest compressions delivered by the medical staff until LDB-CPR was started. We could not evaluate the prehospital or in-hospital quality of conventional CPR with manual chest compression. We began to measure the rSO_2_ of the patients with the NIRS device (TOS-OR; Fujita Medical Co., Tokyo, Japan) when they arrived in the emergency room. Patients underwent LDB-CPR by mechanical chest compression delivered by the AutoPulse^TM^ system (ZOLL, Chelmsford, MA, USA) if they were intubated prior to hospital arrival. Patients who were not intubated at the time of hospital arrival were administered LDB-CPR from 4 minutes after intubation to exclude the effect of oxygenation by intubation. Rhythm analysis and pulse check were performed every second minute. If the rhythm was shockable, defibrillation was performed during LDB-CPR.

The AutoPulse^TM^ system is a portable chest compression device constructed around a backboard that contains a motor that retracts a load-distributing band under microprocessor control. The band is connected to a shaft in the board. The band is tightened and loosened around the chest by the motor, which alternates the rotation of the shaft in both directions. The patient is positioned on the board, and the two broad ends of the band are placed around the patient’s chest and connected to each other. The length of the band automatically adjusts to the size and the shape of the patient. The microprocessor is programmed to provide a constant 20 % reduction in the anterior-posterior dimension of the individual patient’s chest during the compression phase. The compression rate is 80 **±** 5 min-1 with equal periods of compression and unloading, and the device can be operated in a continuous compression mode or in a 15:2 mode. In the 15:2 mode, compressions stop for 3 s after every 15 have been applied to allow two ventilations to be given to the patient. In this study, all resuscitation attempts were performed in the continuous compression mode. LDB-CPR treatment was continued until ROSC or death was declared.

An NIRS device was prepared for operation before patient arrival at the hospital. Upon patient arrival, the patient’s skin was thoroughly cleaned, and the sensor was carefully applied to the patient’s forehead. The rSO_2_ values stabilized within several seconds after placement of the NIRS probes. The rSO_2_ values were then monitored during CPR, and the values measured at each of the points the pulse was checked were used for analysis. The normal range of cerebral rSO_2_ was determined to be over 60 % for the following reasons. First, the oxygen saturation of arterial blood is nearly 100 % and that of venous blood is 50-60 %. Assuming that the arterial blood occupies about one fourth of the tissue blood volume, the mean oxygen saturation in the tissue is estimated to be 60-70 %. When the arterial blood supply is interrupted, oxygen is consumed and the rSO_2_ drops to that of venous blood, around 60 %, and anaerobic metabolism begins. The rSO_2_ actually shows a biphasic decline when arterial perfusion is interrupted: rSO_2_ initially drops rapidly, and then the slope becomes more gradual. Second, the lower normal limit of oxygen saturation that is commonly accepted in monitoring of jugular vein blood is 55 %. Third, the lower limit of oxygen saturation of mixed venous blood is 60 %. Fourth, the range of cerebral rSO_2_ from 15 volunteers (43.2 ± 8.9 years, 10 men and 5 women) breathing room air was 71.2 ± 3.9 %. Finally, a cardiovascular surgery study suggested that the occurrence of neurological events increased when a drop in rSO_2_ of below 60 % was sustained for a long period in elderly people [10]. Clinical staff performed routine post-cardiac arrest interventions regardless of these measurements. All patients were treated principally according to the strategy of the Japan Resuscitation Council 2010 Guidelines.

### Data Collection

After the patients were entered into the study, their rSO_2_ values were measured until ROSC, or LDB-CPR was stopped. The variables of age, sex, and time from the emergency call to arrival at hospital were assessed. We evaluated patient rSO_2_ values pre-LDB-CPR and at 4, 8, and 12 min after the start of LDB-CPR for any cause. Neurological prognosis was evaluated with the Cerebral Performance Category (CPC) score. The presence of any serious adverse events related to LDB-CPR was recorded.

### Statistical Analysis

The comparison of rSO_2_ values was analyzed by analysis of variance adjusted for the pre-rSO_2_ values as a covariate and by the Dunnett test. A *p* value of less than 0.05 was considered to indicate statistical significance. Statistical analyses were performed with JMP Pro version 10.0.2 for Macintosh (SAS Institute Inc., Cary, NC, USA).

## Results

During the study period, 48 consecutive patients fulfilled the inclusion criteria, but 8 patients with ROSC before LDB-CPR, 2 patients with a “Do Not Attempt Resuscitation” order, and 4 patients with difficulties in recording with the NIRS device were excluded. Thus, the data of the remaining 34 patients were analyzed. Table 1 shows the demographic characteristics of the 34 patients, whose mean age was 75.6 ± 12.8 years. Almost all of the patients suffered unwitnessed cardiac arrest at home or at a nursing home/assisting living center. The duration of time from the emergency call to patient arrival at hospital was 31.0 ± 11.4 min. Eight patients were intubated prior to hospital arrival. Of the 34 patients, 13 achieved ROSC (Table 1).

**Table 1:** Patient characteristics

Characteristic	Total (n=34)
Age, mean±SD, years	75.6±12.8
Sex, n (male/female)	21/13
Bystander/witness status, n	
No witness	8
No bystander with witness	10
Family members	5
Others	11
Initially documented rhythm at scene of cardiac arrest, n	
VF/pulseless VT	4
PEA	15
Asystole	15
Time from call to hospital arrival, mean±SD, min	31.0±11.4
Intubation by EMS personnel, n	8
Rhythm at rSO_2_ measurement, n	
VF/pulseless VT	1
PEA	12
Asystole	21
Return of spontaneous circulation, n	13

VF: ventricular fibrillation; VT: ventricular tachycardia; PEA: pulseless electrical activity; EMS: emergency medical services; rSO_2_: regional cerebral oxygen saturation.

**Fig. 1:**
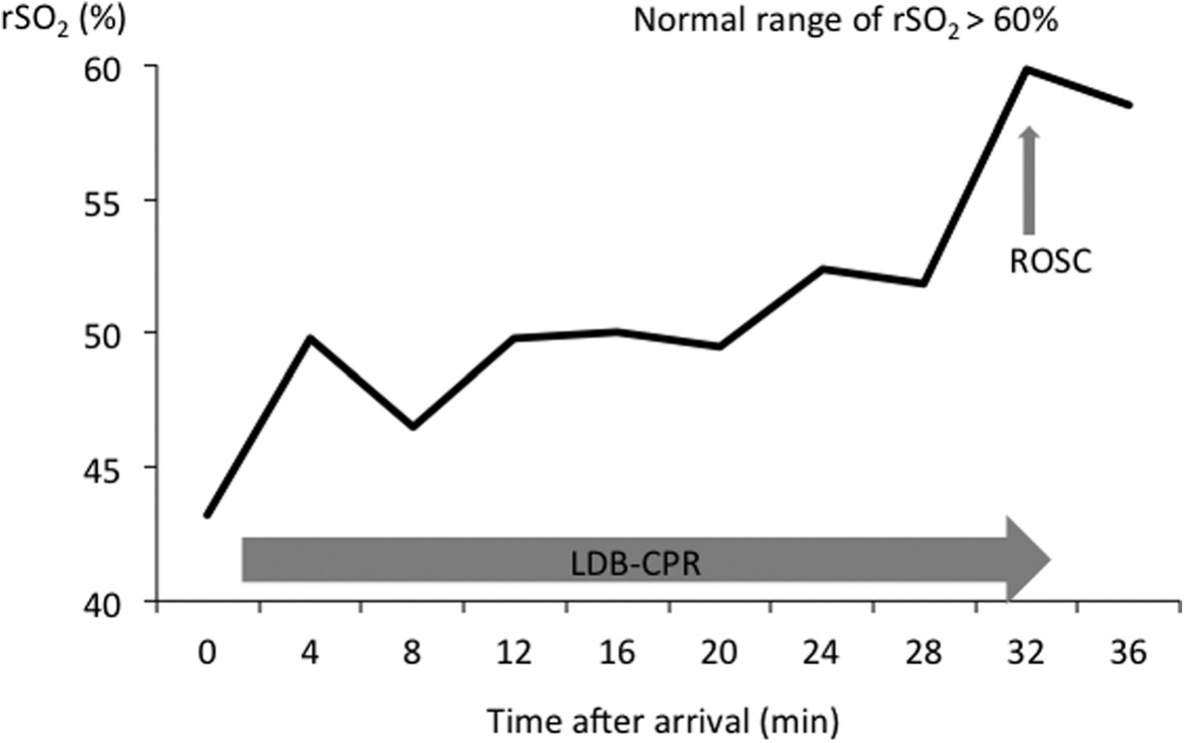
A typical example of the change in rSO_2_ values in a patient with ROSC after LDB-CPR. The rSO_2_ value increased during LDB-CPR and was maintained. After ROSC, the rSO_2_ value continued to increase. rSO_2_: regional cerebral oxygen saturation; ROSC: return of spontaneous circulation; LDB-CPR: load-distributing-band cardiopulmonary resuscitation.

A typical example of the change in rSO_2_ values upon achieving ROSC after LDB-CPR is shown in Fig. 1. The rSO_2_ value increased during LDB-CPR and was maintained, and after ROSC, the rSO_2_ value increased further.

**Fig. 2:**
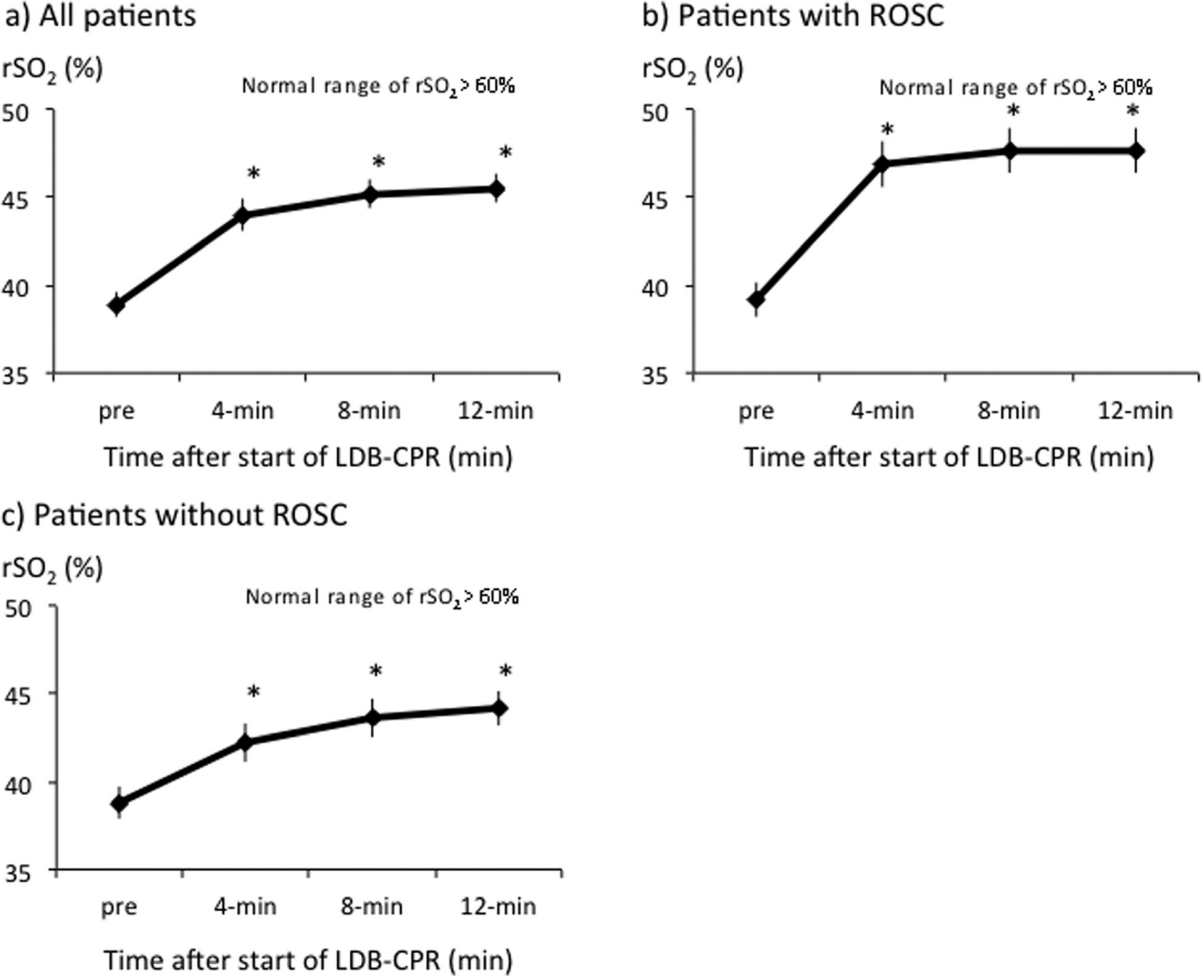
Serial changes in rSO_2_ values during LDB-CPR in a) all patients, b) patients with ROSC, and c) patients without ROSC. Data are expressed as group means ± standard error of the mean. Comparisons of variables between groups were analyzed by analysis of variance adjusted for the pre-rSO_2_ values as a covariate, and *p* values for main treatment effect between groups indicated. *Denotes statistically significant (*p* < 0.05) difference between groups. rSO_2_: regional cerebral oxygen saturation; LDB-CPR: load-distributing-band cardiopulmonary resuscitation; ROSC: return of spontaneous circulation.

Before the start of LDB-CPR, the mean rSO_2_ value among the patients was 38.9 ± 0.7 %. Thereafter, the rSO_2_ value increased significantly at 4, 8, and 12 min after the initiation of LDB-CPR (44.0 ± 0.9 %, 45.2 ± 0.8 %, and 45.5 ± 0.8 %, respectively, p < 0.05) (Fig. 2a). Analysis showed that the rSO_2_ value increased significantly in the patients who achieved ROSC in comparison with those who did not achieve ROSC (Fig. 2b, c).

Of the 13 patients with ROSC, only one patient survived to hospital discharge with a CPC score of 2. The remaining 12 patients either died or survived to hospital discharge with a CPC score of 5. There were no complications caused by LDB-CPR in this study.

## Discussion

In this study, LDB-CPR increased rSO_2_ values compared with manual CPR, and rSO_2_ values were maintained during LDB-CPR. In our previous study, manual chest compression could not increase rSO_2_ without ROSC [9]. Halperin et al. reported that LDB-CPR improved hemodynamics over manual CPR and produced pre-arrest levels of cerebral flow with epinephrine in a pig model [11]. Duchateau et al. reported that a significant improvement in hemodynamics was observed during CPR when the AutoPulse^TM^ automated chest compression device was used compared to the manual active compression-decompression CPR in patients with OHCA [8]. We believe that the increase in rSO_2_ values during LDB-CPR was effected by the hemodynamic improvement brought about by the AutoPulse^TM^.

Kämäräinen et al. showed that the rSO_2_ value could not be improved by manual chest compression CPR with a feedback device [12]. In the present study, the rSO_2_ value pre-LDB-CPR, which reflected the value resulting from prehospital manual chest compression, was very low (38.9 ± 0.7 %), indicating that manual chest compression could not increase the rSO_2_ value. Several studies have found an increase in rSO_2_ after ROSC in patients. The rSO_2_ values also increased significantly in our non-ROSC patients, which may mean that LDB-CPR has effects that increase the rSO_2_ without affecting ROSC.

Hock Ong et al. reported that a resuscitation strategy using LDB-CPR in an emergency department environment was associated with improved neurologically intact survival on discharge in adults who experienced non-traumatic cardiac arrest [13]. Although it is important to maintain the rSO_2_ value to improve the neurological prognosis, in the present study, the neurological prognosis was very poor. As a possible reason, the time from the emergency call to hospital arrival was very long. This could be a result of the long distances and times experienced in transferring the patients living in the mountainous regions or high-rise condominiums. The changes in rSO_2_ values may relate to transfer distance or time. Because the rSO_2_ value was maintained by LDB-CPR, the neurological prognosis might have been improved if LDB-CPR had been initiated in the prehospital setting. We have just begun to study the prehospital changes in rSO_2_ values to help resolve this important problem.

In the CIRC trial, compared to manual CPR, LDB-CPR resulted in statistically equivalent survival and neurological prognosis to hospital discharge after OHCA of presumed cardiac origin [14]. The different findings between the present study and the CIRC trial could be influenced by the differences in the strategy of the resuscitation guidelines. The Japan Resuscitation Council 2010 Guidelines recommend manual CPR only. In the present study, 8 patients were intubated prior to hospital arrival. Although LDB-CPR in patients who were not intubated at the time of hospital arrival was begun from 4 minutes after intubation to exclude the effect of oxygenation by intubation, it is possible that the rSO_2_ values were increased by intubation. It is thus necessary to evaluate rSO_2_ values without intubation between manual CPR and LDB-CPR.

The use of mechanical chest compression devices for CPR remains controversial. A previous meta-analysis showed that the ability to achieve ROSC with mechanical chest compression devices is significantly improved when compared with manual chest compression [15]. In our study, LDB-CPR increased and maintained rSO_2_ values during resuscitation. We think that LDB-CPR may offer a significant beneficial effect on the maintenance of cerebral oxygenation during resuscitation. Moreover, we have developed a portable NIRS system and conducted a pilot study of the device [16]. This will allow us to evaluate changes in rSO_2_ resulting from prehospital mechanical chest compression.

We recognize several limitations in this study. First, the present study is an observational and not a randomized control study. For obvious reasons, this study could not be blinded, and it was difficult to randomize in the prehospital stage. Therefore, we acknowledge the necessity of comparing the changes in rSO_2_ values between conventional CPR with manual chest compression and LDB-CPR in the prehospital stage. Second, this is a single-center study, and third, the sample size was small. But the present study was a pilot study. It was exploratory and for this reason, the objective was 30 patients included (which has been achieved). The final limitation was that the prehospital or in-hospital quality of conventional CPR with manual chest compression could not be evaluated. There are some systems/devices that do not monitor the quality of CPR. None of these systems have been used in this study. The combination of these limitations might cause multiple unmeasured variables to account for the outcome differences observed in this study.

## Conclusion

LDB-CPR significantly increased the rSO_2_ of patients with OHCA. LDB-CPR may have an important beneficial effect on the maintenance of cerebral oxygenation during resuscitation.
